# Case report: Multicomponent intervention for severe food selectivity in autism spectrum disorder: a single case study

**DOI:** 10.3389/fpsyt.2024.1455356

**Published:** 2024-11-11

**Authors:** Roberta Maggio, Laura Turriziani, Silvana Suraniti, Maria Graziano, Santina Patanè, Anna Maria Randazzo, Claudio Passantino, Marcella Di Cara, Angelo Quartarone, Francesca Cucinotta

**Affiliations:** ^1^ Center for Autism "Dopo di noi", Barcellona Pozzo di Gotto, Messina, Italy; ^2^ Psychoeducational Service for Children with Autism and Intellectual Disability, Società Cooperativa Sociale "I Corrieri dell'Oasi", Troina, Italy; ^3^ Cooperativa dalla Luna, Bari, Italy; ^4^ Department of Child and Adolescent Neuropsychiatry (DSM-UOCNPIA), Azienda Sanitaria Provinciale Messina, Messina, Italy; ^5^ Istituto di Ricovero e Cura a Carattere Scientifico, Centro Neurolesi Bonino-Pulejo, Messina, Italy

**Keywords:** applied behavior analysis, feeding disorders, food selectivity, autism spectrum disorder, rehabilitation, intervention, individualized treatment

## Abstract

Food selectivity is common in children with Autism Spectrum Disorder (ASD). The treatment used can be invasive and difficult to implement, necessitating the exploration of multicomponent approaches. This study presents the case of a 9-year-old autistic girl with severe food selectivity, who ate exclusively liquid and semi-solid foods. A multicomponent intervention protocol was developed, including stimulus fading and positive reinforcement techniques, to increase acceptance of new textures and foods. Treatment sessions showed significant improvement in acceptance of semi-solid and novel foods, with a reduction in problem behaviors associated with mealtime. This study suggests that a multicomponent intervention can significantly improve food acceptance and reduce mealtime distress, proving to be a practical and effective treatment strategy in an autistic child. The intervention led to an increase in food acceptance and a reduction in mealtime-related distress, potentially improving the child and family’s quality of life.

## 1 Introduction

Food selectivity in children is a common issue that can have significant impacts on health and well-being (1). Studies indicate that about 13%–50% of typically developed children who experienced feeding difficulties (2, 3). Although there is no single, universally accepted definition of food selectivity, it generally refers to the consumption of a limited range of foods, which often consists of the exclusion of entire food groups, which can lead to nutritional deficiencies and negatively impact growth and development (4, 5). This highly restrictive eating behavior is one of the most commonly reported challenges in Autism Spectrum Disorder children (ASD; 6, 7). Indeed, food selectivity is reported in up to 17-83% of this population, making it one of the most prevalent feeding issues (8–10). Children with food selectivity may restrict their intake to preferred textures or flavors, further compounding nutritional concerns. This condition also creates significant challenges for parents and caregivers, as it may lead to heightened anxiety around mealtimes and can be associated with social withdrawal (11, 12). Moreover, autistic subjects may show increased sensory sensitivity (13), rigid preferences, and difficulty adapting to new foods or textures (14), making the treatment of food selectivity in this population even more difficult (15, 16). Eating difficulties associated with ASD can amplify nutritional and behavioral problems, requiring targeted therapeutic approaches that are sensitive to individual needs (17). Traditional strategies to address food selectivity in children with ASD often rely on techniques like escape extinction, where rejected foods are continuously presented to the child until they accept them (18). Although this approach can be effective, it is frequently associated with high levels of stress for the child and may result in negative experiences around food, potentially increasing food avoidance (19). Given these limitations, recent research has explored more positive, less intrusive interventions aimed at addressing food selectivity in ASD. These include approaches such as food chaining, stimulus fading, and reinforcement-based strategies, which focus on gradually increasing food variety while minimizing distress (20, 21). For instance, newer studies have shown that positive reinforcement and systematic desensitization —where children are gradually exposed to new foods in a supportive and low-pressure environment— can improve food acceptance while reducing the stress associated with4 eating (21, 22). Another promising approach is parent-led interventions that involve caregivers in structured programs to introduce new foods using strategies tailored to the child’s specific sensory preferences and aversions. These methods have demonstrated both acceptability to families and effectiveness in increasing dietary variety intake (20, 22). One promising approach is the use of antecedent manipulations, which involve modifying the environment or circumstances preceding the meal to make the eating experience more positive and acceptable to the child (23). Another effective methodology is positive reinforcement, which incentivizes desired behavior through rewards and reinforcement, rather than punishment or pressure (5). These approaches aim to create a more supportive environment for the child, promoting acceptance of new foods through positive experiences and gradual supports (24). Our case report aims to expand the existing body of knowledge by introducing a treatment package combining various protocols and techniques. Specifically, our approach integrates the protocol on texture acceptance of certain foods with handling and fading, as well as the use of “non-taste exposure techniques” to promote the acceptance of novel foods. The strategy also includes the use of shaping and simultaneous and sequential food presentation to increase acceptance of both consistency and newly introduced foods. The main objective of this study is to demonstrate the effectiveness of these combined methodologies as viable alternatives to the use of escape extinction. It is hypothesized that such approaches may be particularly beneficial for some children with food selectivity, including those with ASD, offering a first line of treatment that can be easily implemented by parents, teachers, and clinicians with limited training. To our knowledge, to date, only few studies have evaluated an approach based on a multicomponent intervention in the treatment of food selectivity in children with ASD (25). This could be an important step toward more specialized interventions to improve the quality of life for children and their families.

## 2 Case presentation

The participant is a 9-year-old girl diagnosed with Autism Spectrum Disorder (ASD), requiring Level 3 support. On the Leiter International Performance Scale – Third edition (26), which assesses nonverbal intelligence, she scored a nonverbal IQ of 54. The Vineland Adaptive Behavior Scales - Second edition (27) revealed a low level of adaptive functioning in all areas: communication, daily living skills, and socialization, with equivalent scores at ages 1 to 3 years and a deviation IQ score of 86. On the Verbal Behavior Milestones Assessment and Placement Program (VB-MAPP) (28), she scored 42, with a heterogeneous profile at level 1, showing significant deficits in the skills of social behavior, social play, and motor imitation. The child presents echolalia and low functional use of language.

### 2.1 Method

An indirect assessment revealed the participant’s feeding difficulties. It was found that, for more than 9 years, the child had been consuming liquid and semi-solid foods such as homogenized baby food, no more than 5mm diameter mash with specific shape (stars), and smoothies, never having meals outside the home and always being fed by her mother. Before the intervention conducted at the Day Care Center, the child had undergone private treatment where attempts were made to introduce foods not typically consumed by her, with little success, as she refused these types of foods and exhibited significant problem behaviors, including self-harm. After initial intake and a detailed medical and speech evaluation, conducted by a chewing and swallowing specialist within a multidisciplinary team, the decision was made to intervene. The initial goal was to increase the repertoire of foods consumed and improve the consistency to a full-bodied, semi-solid compound. Subsequently, further evaluation is planned to assess oral-buccal movements and swallowing in order to assess the possibility of introducing solid foods that the child can chew. The target behavior of the intervention was 100% acceptance of the consistency of semi-solid foods and 100% acceptance of new foods, without manifesting problem behaviors. For this purpose, 3 solid foods and 3 new foods were introduced for consumption. The target food was considered acquired when the participant achieved 100% acceptance for at least two consecutive sessions, without manifesting problem behaviors.

The primary dependent variable of the study was the acceptance and amount of foods with increased texture and firmness. The second dependent variable was the acceptance and inclusion of new foods in the participant’s food repertoire. During treatment, detailed data were collected on each single trial of food presented to the child, assessing the percentage of food accepted without emergence of problem behaviors for at least two consecutive sessions. Only after this criterion was met for a particular food was the next food introduced into the treatment program. All sessions were filmed in order to be able to record data correctly in the cards without incurring error, for the purpose of later reviewing the videos and calculating inter-observer agreement (IOA). IOA was calculated by dividing the number of agreements by the total number of agreements plus disagreements, and then multiplying by 100 (29). In all intervention sessions, the calculated IOA was 95%.

The present study was conducted following an AB-type experimental design, in which phase A represents the baseline and phase B represents the intervention. This type of design can demonstrate a correlation between the independent and dependent variables. Two different procedures had to be carried out to work in parallel on the acceptance of new textures and novel foods (1) Stimulus Fading and Texture procedure and (2) Simultaneous and sequential shaping and presentation of foods. Both procedures were integrated within the same daily schedule, allowing for consistent and structured exposure to new textures and foods. This routine ensured that both procedures were carried out within a 3-hour treatment window, 4 days a week, with dedicated time for each during the sessions. During these sessions, the Stimulus Fading and Texture procedure was performed first, followed by other rehabilitative activities, after which the Simultaneous and Sequential Shaping and Presentation of Foods session took place.

### 2.2 Intervention

#### 2.2.1 Stimulus fading and texture fading

Before the intervention, a weekly analysis of the participant’s daily diet (breakfast, lunch, snack, dinner) was conducted to identify favorite foods. Data collection was included for every single trial presented to the participant regarding swallowing the foods. The percentage of food acceptance without the occurrence of problem behaviors was calculated over two consecutive sessions before moving on to the next diet. During snack time, a baseline was performed for each combination of foods, which included cream with sponge cake, fruit with yogurt, and vanilla pudding. For each food combination, the participant’s acceptance of the texture and flavor was observed, and the occurrence of any problem behaviors was recorded to establish a baseline of her current eating patterns. The experimenter presented the discriminative stimuli (SD), which are presentation of food (e.g., liked food + crumbs) or a verbal prompt (e.g., “you can eat”). In the first case, liked foods with small amounts of less preferred textures were presented without providing feedback or reinforcement. Data were collected from two trials for each target for at least 2 consecutive days. The procedure of Stimulus Fading, which involves gradually introducing a less preferred or more challenging stimulus (in this case, solid or semi-solid foods) by combining it with a more preferred or easier stimulus, and Texture Fading (30) was implemented. This was done by gradually introducing liked creamy foods (fruit yogurt, vanilla pudding, cream) mixed with less liked solid and semi-solid foods (sponge cake, fruit, brioche). The treatment was performed during snack time, using a chair, a table, a teaspoon and a saucer. The introduction began with 70% liquid/creamy foods + 5 grams of solids, gradually reducing the liquids and increasing the solids until a semi-solid, nutritious consistency was achieved. During each session, one target food was presented at a time (e.g., cream + sponge cake), following the established hierarchy of textures, which refers to the gradual progression from easier-to-swallow, smoother foods (such as purees or creams) to more challenging textures (such as semi-solids and solids like sponge cake or fruit). This hierarchy helps the participant adapt to more complex food textures over time. In other instances, the experimenter provided the SD “you can eat,” waiting for the participant to ingest the food within 5 seconds and consume the entire bite. If the food was refused or spit out, the spoon was removed without providing feedback. Next, the SD with the percentage of food from the previous step was presented again, waiting for the participant to agree to ingest the target food. Once the food was accepted and two consecutive sessions were completed without any problem behaviors, the next step was taken. The final target was achieved with the acceptance of 10% liquids + 35 grams of solids.

#### 2.2.2 Shaping, simultaneous and sequential presentation of foods

Before starting the second intervention protocol, an analysis of foods not consumed daily by the participant was conducted to expand her food repertoire. This evaluation was based on data collection focused on foods that the participant did not typically ingest due to their creamy texture. The goal was to introduce new foods to be consumed daily. Specifically, mashed potatoes, plain yogurt, and cheese spread were selected as target foods due to their creamy consistency and because they represented a significant texture difference from the foods the participant regularly consumed. These foods were chosen because they were less preferred by the participant, and their introduction would help in expanding her tolerance to a wider variety of textures. A baseline was conducted for these foods, which the experimenter presented the SD (disliked food near the lips) along with the instruction to taste, without providing feedback or reinforcement. After tasting, the participant was given 3 seconds to respond. Data were collected over ten trials for each target for at least 2 consecutive days. The foods were introduced in small, manageable quantities to facilitate gradual acceptance, focusing not only on texture but also on nutritional value (such as cheeses). The program used shaping sessions, a behavioral technique that combines extinction and reinforcement to encourage variability and produce new behaviors based on that variability. This method involves reinforcing successive approximations of the target behavior. In this case, with simultaneous and sequential presentation of foods (31), following a hierarchy of six steps designed to help the participant gradually accept disliked foods: visual tolerance, interaction without touching, smelling, touching, tasting, and finally eating. The treatment began during a sensory session where the participant was encouraged to manipulate tactile materials and foods she liked (e.g. boiled pasta, sawdust, flour mixed with water). Materials used in the session included chair, table, bowl, saucer, and a portion of disliked food (5 teaspoons) such as mashed potatoes, spreadable cheese, or plain yogurt. These foods were selected based on their creamy texture, which provided a progression from the participant’s existing preferred foods and allowed for gradual texture fading. The simultaneous and sequential presentation of foods followed a hierarchy of six steps aimed at gradually increasing acceptance of disliked foods: visual tolerance, interaction without touching, smelling, touching, tasting, and finally eating. For each target food (e.g., spreadable cheese), the experimenter followed the structured protocol. After the participant successfully completed step 4 (touching the food), step 5 was initiated. Here, the child was allowed to manipulate the preferred sensory materials for at least 5 minutes. Then, the experimenter presented the SD “can you taste” and made the teaspoon of disliked food available, waiting for the participant to taste the food within 5 seconds. If the participant exhibited the target behavior (e.g., tasting the food), it was reinforced by allowing further interaction with the preferred sensory materials. This sequence was repeated until the entire portion of food (5 teaspoons) was consumed. In case where the participant of rejection the disliked food, the teaspoon was withdrawn without providing feedback. The SD from Step 4 was then re-presented, and once the participant touched the food, praise was provided. The process continued, and after the participant tolerated 100% of the bites in Step 6 (eating the new food) for two consecutive sessions, the next step was introduced. The ultimate goal of the intervention was achieved when the participant successfully accepted three new foods.

## 3 Result

**Figure 1:**
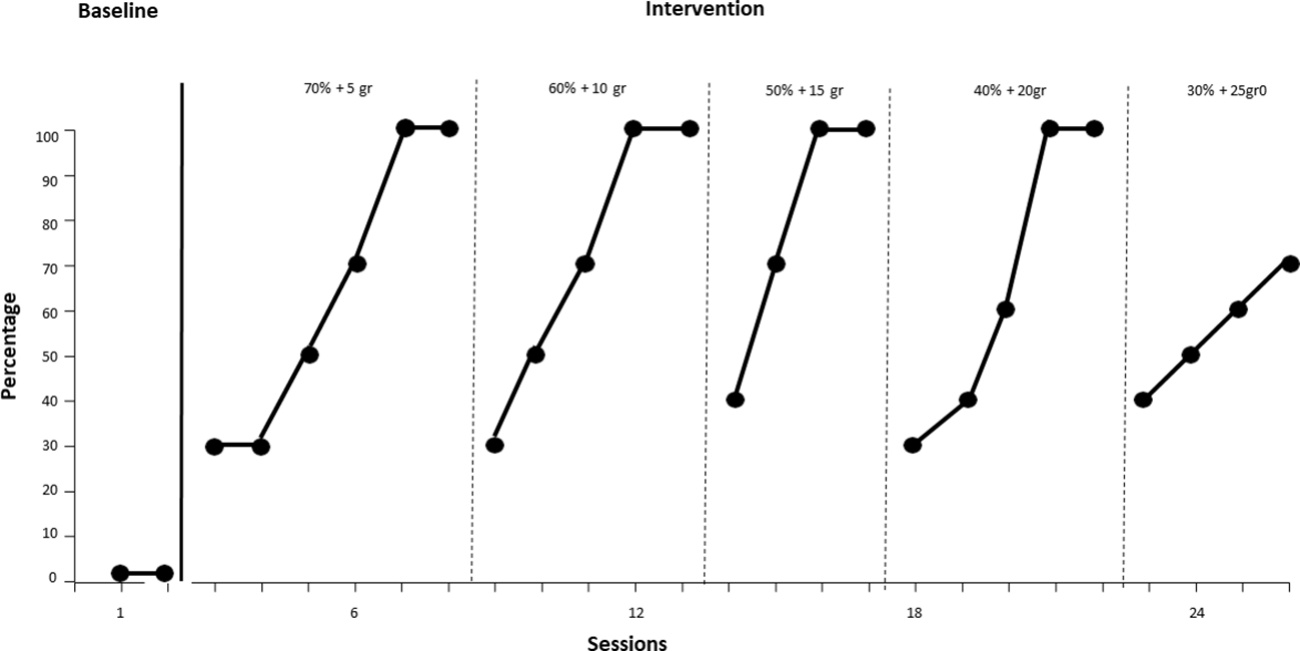
Intake of "Creamy Plus Sponge Cake" over time.

The results obtained show a correlation between the independent and dependent variables as can be seen in the figures below. A Changing Criterion Design was used for the first procedure, which evaluates the effects of treatment when applied in a stepwise or phased manner on a target behavior. After the initial baseline, the treatment was divided into steps with different criteria within, each step being closer to the final behavioral goal of ingesting a lower percentage of liquid/creamy food and a higher percentage of solid food. Three foods specifically were evaluated (cream + sponge cake/fruit + yogurt/vanilla pudding+ brioche). In **Figure 1**, it is observed that during baseline, the participant shows no intake of “cream plus sponge cake,” maintaining a steady trend to zero in the first two sessions. With the start of treatment from session 3 to session 26, an upward trend is shown: from session 3, with 30% intake of 70% liquid/creamy foods + 5 grams of sponge cake, until reaching 100% in session 8. For the criterion of 60% liquid/creamy foods + 10 grams, intake increases from 30% in session 9 to 100% in session 13. The behavior remains stable from session 14 to 22, maintaining a constant consistency. In sessions 23-26, although the trend is upward, the full target is not reached, remaining at 70% proficiency. Therefore, the treatment continues to reach the desired target.

**Figure 2:**
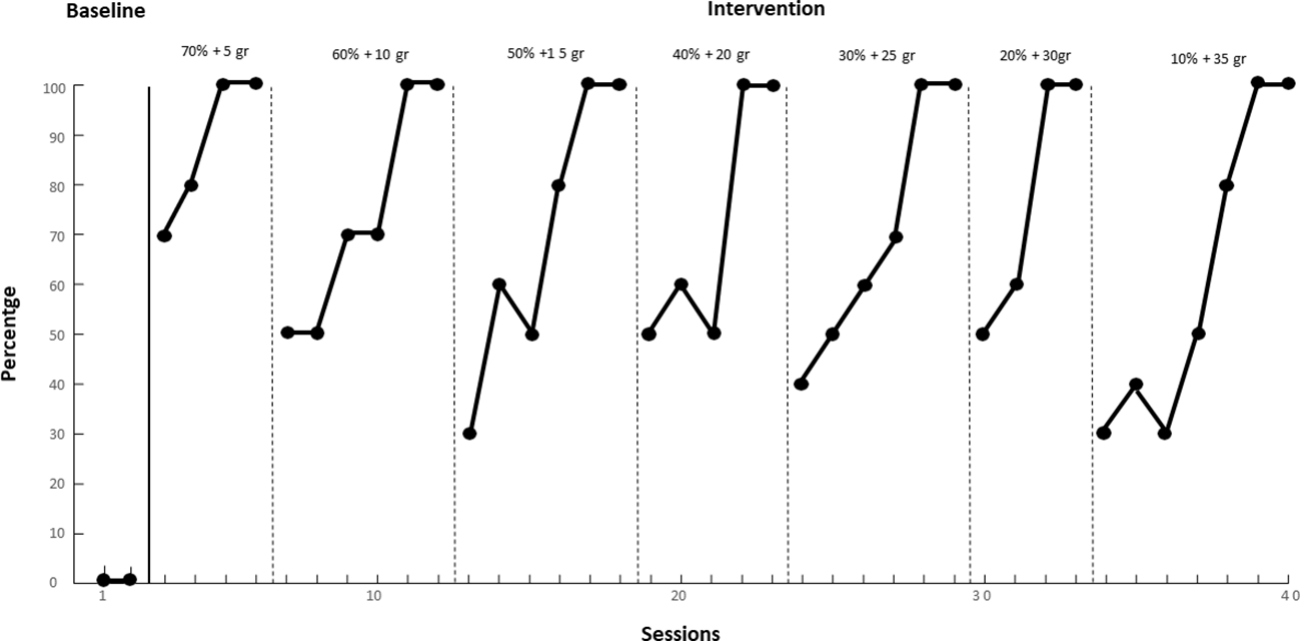
Intake of "Yogurt and Fruit" over time.

In **Figure 2**, during baseline, the participant shows a steady zero trend in the first two sessions. With the start of treatment from session 3 to session 7, an upward trend is observed: for the yogurt and fruit mixture with 70% liquid/creamy and 5 grams of solids, the target behavior increases from 70% to 100%. Moving to the next criterion of 60% liquids/creamy + 10 grams solids up to 20% liquids/cremates and 30 grams solids, the trend remains upward and stable throughout the intervention sessions (session 7 to session 33), reaching 100% of food ingested. This composition was identified as highly palatable during treatment. From session 34 to session 40, with the food composed of 10% liquids/creamy and 35 grams of solids, the trend continues to be upward, starting from 30% of bites ingested and reaching 100%. The behavior of eating “yogurt and fruit” remains constant at 100%, thus achieving the established acquisition criterion.

**Figure 3:**
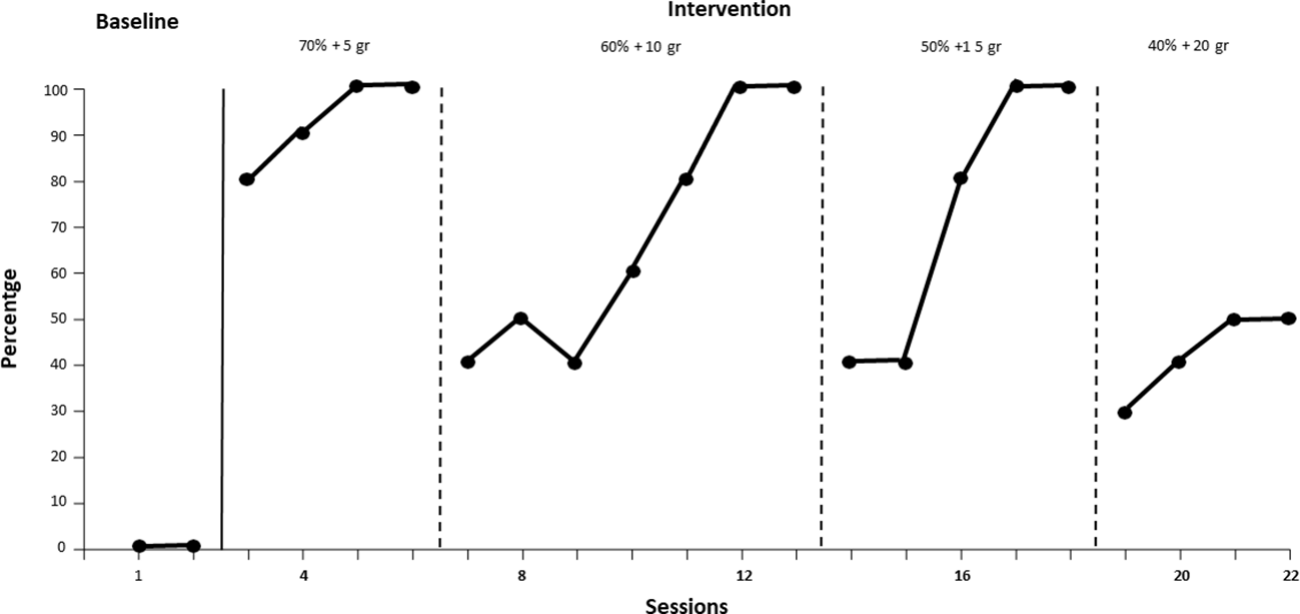
Intake of "Vanilla Pudding with Brioche" over time.

In **Figure 3**, during baseline, the participant shows a steady trend to zero in the first two sessions for the behavior of eating “vanilla pudding with brioche.” With the start of treatment from session 3 to session 6, an upward trend is observed: from 80% to 100% of a compound with 70% liquids/creams and 5 grams of solids. From session 7 to session 18, with the goal of eating from 60% liquids/creamy + 10 grams up to 50% liquids/creamy + 15 grams solids, the behavior fluctuates from the initial difficulty of 40% of mouthfuls swallowed up to the achievement of 100%, thus achieving the acquisition criterion. During the treatment, 100% intake was achieved for brioche pudding in the stage with 40% liquids/creamy and 20 grams of solids, with 50% bites ingested. Therefore, it is necessary to continue the treatment for this food as well.

**Figure 4:**
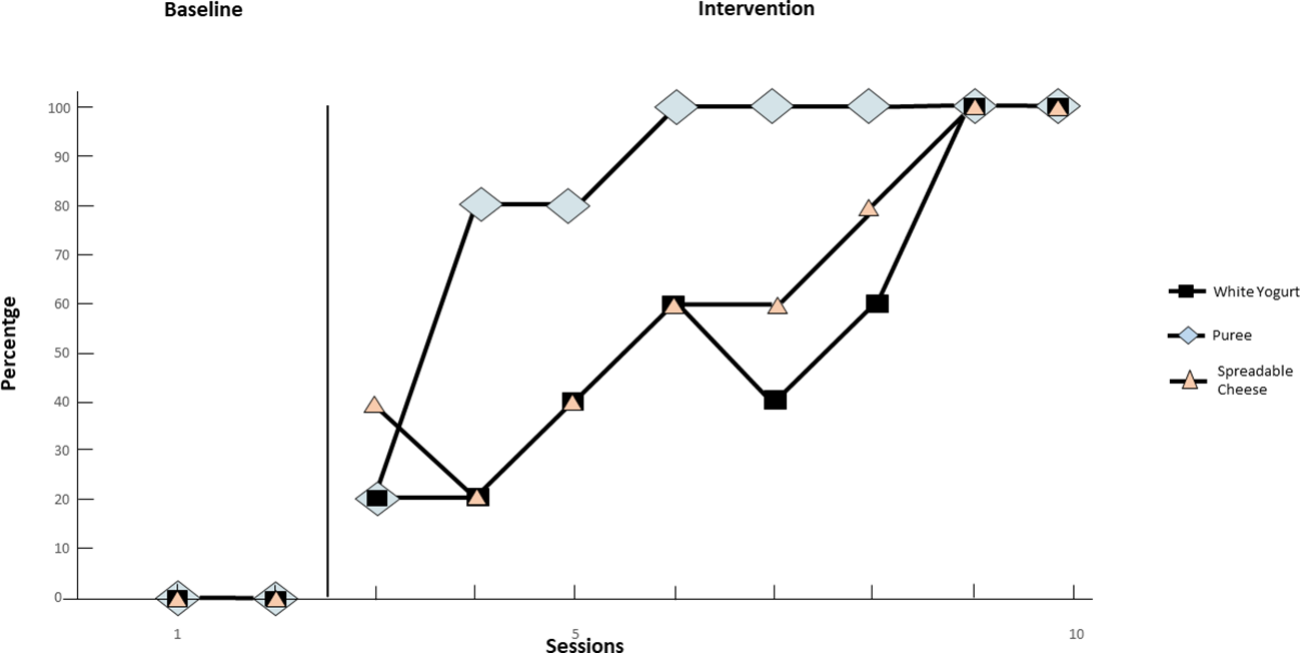
Intake of new foods (White Yogurt, Spreadable Cheese, Puree) over time.

In **Figure 4**, during the baseline, the participant shows a steady zero trend in the first two sessions for the behavior of eating new foods such as white yogurt, spreadable cheese, and puree. With the onset of treatment from session 3 to session 10, an upward trend is observed: at session 6, the participant shows 60% eating behavior for yogurt and cheese, while reaching 100% target behavior in eating puree. The acquisition criterion for mashed potato is reached earlier, in sessions 6-7, while for yogurt and spreadable cheese it is reached in session 9. In the next three sessions (sessions 9 to 10), the behavior remains constant at 100%, thus reaching the established acquisition criterion.

## 4 Discussion

The treatment of food selectivity in ASD represents a significant challenge for families and professionals (32). The prevalence of feeding issues in this population is well documented and can lead to serious nutritional and psychological consequences (4). Many traditional approaches focus on single techniques, which are often intrusive and difficult to apply in everyday settings (5). The present study sought to develop a multicomponent protocol that integrates several intervention strategies to improve acceptance of new foods and textures while reducing discomfort associated with eating (17). This approach aims to provide a more comprehensive and easily implemented solution, potentially improving the quality of life for children with ASD and their families (33). The results found in the present study highlight the effectiveness of a combined protocol integrating antecedent manipulation, positive reinforcement, and texture fading with non-taste exposure and shaping techniques. This approach has been shown to be particularly effective in increasing the acceptance of foods with new textures and the introduction of new foods in ASD. These results are significant not only because of the individuality of the case, but also because of the broader implications in the treatment of food selectivity in children with ASD.

This case report offers valuable insight into the potential effectiveness of multicomponent intervention. Unlike many previous studies that focus on a single technique, our protocol integrates several intervention methodologies. The combined use of texture fading, antecedent manipulation, positive reinforcement, and non-taste exposure techniques has allowed us to comprehensively address issues related to food selectivity. This approach is supported by research that emphasizes the effectiveness of multiple techniques combined to improve food acceptance in children with ASD (5, 34). Moreover, traditionally, escape extinction has been a common technique to address food selectivity. However, this can be stressful and difficult to apply (5). In contrast, our approach uses less intrusive and more positive techniques, reducing potential discomfort for the child and facilitating implementation by parents and caregivers with limited training. Recent studies have demonstrated the effectiveness and acceptability of positive approaches in managing food selectivity (35). In addition, the developed protocol can be easily adapted and applied in different settings, including home and school environments. This makes the intervention accessible to a wider range of families and professionals, potentially improving the quality of life for many children with ASD. The versatility and ease of implementation of such interventions have been highlighted in the literature (36). The data collected show a clear upward trend in the acceptance of new foods and textures, with stable maintenance of the target behavior once achieved. This confirms the effectiveness of the protocol not only in the short term, but also in maintaining the progress made. Long-term effectiveness and stability of the acquired eating behavior are crucial aspects of the intervention (37, 38). The data collected show a clear upward trend in the acceptance of new foods and textures, with stable maintenance of the target behavior once achieved. This confirms the effectiveness of the protocol not only in the short term but also in maintaining the progress made. Despite the promising results, the study has some limitations.

The research focused on a single case, and it is important to interpret these results with caution. Furthermore, the results may not be generalizable to all ASD individuals: future studies should include larger samples to validate the effectiveness of this protocol. Moreover, it will be necessary to evaluate several clinical elements that may play a moderating role on outcome and on therapeutic intervention processes, such as intelligent quotient levels, ASD severity, and family compliance. Finally, feasibility in community settings seems to be an unexplored issue, necessarily to translate research findings in clinical practice. In addition, it would be useful to explore the long-term impact of the intervention and possible variables that might influence outcomes, such as the level of family support and individual differences in sensory profiles (11).

## 5 Conclusion

Treatments of pediatric feeding disorders based on applied behavior analysis have the most empirical support in the research literature (39). Our findings contribute to presenting a promising approach for food selectivity in children with ASD. The integration of different intervention techniques is effective, less intrusive, and easily applicable in a variety of settings. The results not only expand the existing literature but also offer new directions for clinical practice and future research.

## Data Availability

The raw data supporting the conclusions of this article will be made available by the authors, without undue reservation.

## References

[1] Hubbard KL Anderson SE Curtin C Must A Bandini LG . A comparison of food refusal related to characteristics of food in children with autism spectrum disorder and typically developing children. J Acad Nutr Diet. (2014) 114:1981–7. doi: 10.1016/j.jand.2014.04.017 PMC4252256 24928779

[2] Luisier A-C Petitpierre G Clerc Bérod A Garcia-Burgos D Bensafi M . Effects of familiarization on odor hedonic responses and food choices in children with autism spectrum disorders. Autism. (2019) 23:1460–71. doi: 10.1177/1362361318815252 30523698

[3] Schmitt L Heiss CJ Campbell EE . A comparison of nutrient intake and eating behaviors of boys with and without autism. Top Clin Nutr. (2008) 23:23–31. doi: 10.1097/01.tin.0000312077.45953.6c

[4] Bandini LG Anderson SE Curtin C Cermak S Evans EW Scampini R . Food selectivity in children with autism spectrum disorders and typically developing children. J Pediatr. (2010) 157:259–64. doi: 10.1016/j.jpeds.2010.02.013 PMC2936505 20362301

[5] Sharp WG Burrell TL Jaquess DL . The Autism MEAL Plan: a parent-training curriculum to manage eating aversions and low intake among children with autism. Autism. (2014) 18:712–22. doi: 10.1177/1362361313489190 24101716

[6] Cermak SA Curtin C Bandini LG . Food selectivity and sensory sensitivity in children with autism spectrum disorders. J Am Diet Assoc. (2010) 110:238–46. doi: 10.1016/j.jada.2009.10.032 PMC3601920 20102851

[7] Sharp WG Berry RC McCracken C Nuhu NN Marvel E Saulnier CA . Feeding problems and nutrient intake in children with autism spectrum disorders: a meta-analysis and comprehensive review of the literature. J Autism Dev Disord. (2013) 43:2159–73. doi: 10.1007/s10803-013-1771-5 23371510

[8] Alibrandi A Zirilli A Loschiavo F Gangemi MC Sindoni A Tribulato G . Food selectivity in children with autism spectrum disorder: A statistical analysis in southern Italy. Children. (2023) 10. doi: 10.3390/children10091553 PMC10527699 37761514

[9] Sharp WG Berry RC McCracken C Nuhu NN Marvel E Saulnier CA . Feeding problems and nutrient intake in children with autism spectrum disorders: A meta-analysis. J Pediatr Psychol. (2020) 44:988–1002. doi: 10.1007/s10803-013-1771-5 23371510

[10] Ledford JR Gast DL . Feeding problems in children with autism spectrum disorders: A Review. Focus on Autism and Other Developmental Disabilities. (2006) 21:153–66. doi: 10.1177/10883576060210030401

[11] Esteban-Figuerola P Canals J Fernández-Cao JC Arija Val V . Differences in food consumption and nutritional intake between children with autism spectrum disorders and typically developing children: A meta-analysis. Autism. (2019) 23:1079–95. doi: 10.1177/1362361318794179 30345784

[12] Seiverling L Williams K Sturmey P . Assessment of feeding problems in children with autism spectrum disorders. J Dev Phys Disabil. (2010) 22:401–13. doi: 10.1007/s10882-010-9206-0

[13] Suarez MA Nelson NW Curtis AB . Longitudinal follow-up of factors associated with food selectivity in children with autism spectrum disorders. Autism. (2014) 18:924–32. doi: 10.1177/1362361313499457 24121181

[14] Esposito M Mirizzi P Fadda R Pirollo C Ricciardi O Mazza M . Food selectivity in children with autism: Guidelines for assessment and clinical interventions. Int J Environ Res Public Health. (2023) 20:5092. doi: 10.3390/ijerph20065092 36982001 PMC10048794

[15] Mayes SD Zickgraf H . Atypical eating behaviors in children and adolescents with autism, ADHD, other disorders, and typical development. Res Autism Spectr Disord. (2019) 64:76–83. doi: 10.1016/j.rasd.2019.04.002

[16] Curtin C Hubbard K Anderson SE Mick E Must A Bandini LG . Food selectivity, mealtime behavior problems, spousal stress, and family food choices in children with and without autism spectrum disorder. J Autism Dev Disord. (2015) 45:3308–15. doi: 10.1007/s10803-015-2490-x PMC4573255 26070276

[17] Marshall J Ware R Ziviani J Hill RJ Dodrill P . Efficacy of interventions to improve feeding difficulties in children with autism spectrum disorders: a systematic review and meta-analysis: Efficacy of feeding interventions in young children with ASD. Child Care Health Dev. (2015) 41:278–302. doi: 10.1111/cch.12157 24962184

[18] LaRue RH Stewart V Piazza CC Volkert VM Patel MR Zeleny J . Escape as reinforcement and escape extinction in the treatment of feeding problems. J Appl Behav Ana. (2011) 44:719–35. doi: 10.1901/jaba.2011.44-719 PMC3251277 22219525

[19] Kuschner ES Eisenberg IW Orionzi B Simmons WK Kenworthy L Martin A . A preliminary study of self-reported food selectivity in adolescents and young adults with autism spectrum disorder. Res Autism Spectr Disord. (2015) 15-16:53–9. doi: 10.1016/j.rasd.2015.04.005 PMC4545503 26309446

[20] Seiverling L Hendy HM Williams KE . A review of food chaining and systematic desensitization interventions for children with ASD. Res Dev Disabil. (2018) 80:103–17. doi: 10.1016/j.ridd.2018.05.006

[21] Patel MR Piazza CC Layer SA Volkert VM . Effects of systematic desensitization on food selectivity in children with autism spectrum disorder. J Appl Behav Anal. (2020) 53:1130–46. doi: 10.1002/jaba.714

[22] Marshall J Ware R Ziviani J Hill RJ Dodrill P . A family-centered feeding intervention for food selectivity in children with ASD: Outcomes and acceptability. J Autism Dev Disord. (2022) 52:2437–52. doi: 10.1007/s10803-021-05150-z

[23] Piazza CC Fisher WW Brown KA Shore BA Patel MR Katz RM . Functional analysis and treatment of chronic food refusal in children with pediatric feeding disorders. J Appl Behav Analysis. (2019) 46:167–76. doi: 10.1901/jaba.2003.36-187

[24] Hyman SL Levy SE Myers SM . Identification, evaluation, and management of children with autism spectrum disorder. Pediatrics. (2020) 145:e20193447. doi: 10.1542/peds.2019-3447 31843864

[25] Peterson KM Piazza CC Volkert VM . A comparison of a modified sequential oral sensory approach to an applied behavior-analytic approach in the treatment of food selectivity in children with autism spectrum disorder. J Appl Behav Anal. (2016) 49:485–511. doi: 10.1002/jaba.332 27449267

[26] Cornoldi C Giofrè D Belacchi C . Leiter-3 Leiter International Performance Scale Tirth Edition. Florence, Italy: Giunti O.S. (2016).

[27] Burger-Caplan R Saulnier CA Sparrow SS . Vineland Adaptive Behavior Scales. In: Encyclopedia of Clinical Neuropsychology Vol. p. . Cham: Springer International Publishing (2018) p. 1–5.

[28] Sundberg ML . VB-MAPP Verbal Behavior Milestones Assessment and Placement Program: a language and social skills assessment program for children with autism or other developmental disabilities: guide. Mark Sundberg. (2008).

[29] Cooper JO Heron TE Heward WL . Improving and assessing the quality of behavioral measurement. In: Cooper JO Heron TE Heward W , editors. Applied Behavior Analysis. Pearson Merrill Prentice Hal, New Jersey (2007). p. 113–6.

[30] Shore B Piazza CC . Manual for the assessment and treatment of the behavior disorder of people with mental retardation. Konarski E Favell J , editors. New York: The Guilford Press (1998).

[31] Weber J Gutierrez A Jr . A treatment package without escape extinction to address food selectivity. J Vis. (2015). doi: 10.3791/52898-v PMC4692558 26325108

[32] Matson JL Fodstad JC . The treatment of food selectivity and other feeding problems in children with autism spectrum disorders. Res Autism Spectr Disord. (2009) 3:455–61. doi: 10.1016/j.rasd.2008.09.005

[33] Bandini LG Curtin C Phillips S Anderson SE Maslin M Must A . Changes in food selectivity in children with autism spectrum disorder. J Autism Dev Disord. (2017) 47:439–46. doi: 10.1007/s10803-016-2963-6 PMC5310968 27866350

[34] Seiverling L Williams K . The role of parental and practitioner variables in the treatment of pediatric feeding problems. Clin Child Family Psychol Review. (2018) 21:81–92. doi: 10.1007/s10567-017-0257-3

[35] Bachmeyer MH Piazza CC . Functional analysis and treatment of feeding disorders. J Appl Behav Analysis. (2019) 52:163–89. doi: 10.1901/jaba.2009.42-641

[36] Johnson CR Handen BL . Effects of a behavioral feeding intervention on caregiver stress in children with autism spectrum disorder. J Autism Dev Disord. (2015) 45:2831–42. doi: 10.1007/s10803-015-2440-7

[37] Koegel LK Koegel RL . Improving feeding and nutrition in children with ASD: A study of behaviorally based approaches. J Autism Dev Disord. (2012) 42:113–23. doi: 10.1007/s10803-011-1392-9

[38] Mueller M Piazza CC . Comparison of two approaches to increase food consumption in children with autism spectrum disorders. J Appl Behav Analysis. (2018) 51:285–301. doi: 10.1002/jaba.446

[39] Volkert VM Piazza CC . Handbook of evidence-based practice in clinical psychol-ogy Vol. 1. Sturmey P Hersen M , editors. Hoboken, NJ: Wiley (2012).

